# Admission Decisions Made by Emergency Physicians Can Reduce the Emergency Department Length of Stay for Medical Patients

**DOI:** 10.1155/2020/8392832

**Published:** 2020-02-11

**Authors:** Yuri Choi, Jinwoo Jeong, Byoung-Gwon Kim

**Affiliations:** ^1^Department of Emergency Medicine, Dong-A University College of Medicine, Busan, Republic of Korea; ^2^Department of Medicine, Graduate School of Dong-A University, Busan, Republic of Korea; ^3^Department of Preventive Medicine, Dong-A University College of Medicine, Busan, Republic of Korea

## Abstract

**Background:**

Emergency department (ED) overcrowding is a worldwide problem that poses a threat to patient safety by causing treatment delays and increasing mortality. Consultations are common and important in the emergency medicine profession and are associated with longer ED length of stay (LOS). The purpose of this study was to evaluate the impact of admission decisions by emergency physicians without consultations on the ED LOS and other quality indicators.

**Methods:**

The study was a retrospective observational study comparing the ED LOS of patients admitted to the internal medicine (IM) department before and after the policy change regarding admission decisions that was implemented in October 2016. During and after the policy change, emergency physicians decided how to arrange for and treat medical patients by processing their admission and providing follow-up care without consultations. The ED LOS and other indicators of patients admitted to the IM department were compared between the study period (January to June 2017) and the control period (January to June 2016).

**Results:**

The median ED LOS of patients admitted to the IM department decreased from 673 (IQR: 347–1,369) minutes in the control period to 237 (IQR: 166–364) minutes in the study period. There were no significant differences in the interdepartmental transfer rate or in-hospital mortality between the two periods.

**Conclusions:**

The admission decisions regarding medical patients made by emergency physicians without specialty consultations reduced the ED LOS without a significant negative effect on mortality or hospital LOS.

## 1. Introduction

Emergency department (ED) overcrowding is a worldwide problem that poses a threat to patient safety by causing treatment delays and increasing mortality [1–3]. A prolonged ED length of stay (LOS) contributes to overcrowding, and its consequences include a decreased quality of treatment, the misuse of medical resources in the ED, and decreased patient satisfaction [3–5]. ED overcrowding has been related to poor clinical outcomes, such as high mortality and morbidity [6,7]. Presenting to an ED during shifts with longer waiting times was associated with a higher risk of death and admission to the hospital in the short term [5,8].

Consultations are common and important in the emergency department [9]. ED consultations are defined as requests for assistance, while the patient remains under the care of the emergency physician [10]. Although there are a variety of factors that affect ED LOS and ED overcrowding, challenges during consultations are considered a major cause of delay in the patient flow [11–14]. Admission decisions made with specialist consultations take a long period of time because physicians in specialty departments outside of the ED are often preoccupied with inpatient or routine services [15]. Common reasons for consultation process delays have been reported to include urgent ward issues, the need for multiple ED consultations, and the need for additional laboratory or radiographic evaluations [13]. The impact of consultations on the ED LOS is high in tertiary-care centers, where the number of ED consultations is reported to be high [11,13,16].

ED consultations are performed to resolve issues regarding hospital admission, to obtain expert opinions, to provide treatments or procedures, and to obtain outpatient referrals [11,13]. Among these reasons for consultations, consultations for issues regarding hospital admission are common and considered mandatory in many systems or centers [11,13,16]. Some authors have tried to optimize the consultation process by implementing new hospital guidelines, computerization, and quality improvement methodologies [2,17–19]. However, it has also been suggested that terminating mandatory consultations will reduce the impact of consultations on the ED LOS [16]. Recently, since the initiation of a special policy to restrict residents' work hours in 2016 and reduce the duration of IM residency from 4 years to 3 years in Korea, the shortage of inpatient medical staff has been a significant concern, especially in the internal medicine (IM) department [20]. One of the countermeasures to address such difficulties was to reduce the involvement of the IM in ED management and disposition decisions. Thus, the mandatory consultation policy was abandoned in our hospital, and the authors of this study aimed to evaluate the impact of admission decisions by emergency physicians without consultations on the ED LOS and other quality indicators.

## 2. Methods

### 2.1. Study Design

The study was a retrospective observational study comparing the ED LOS of patients admitted to the IM department before and after the policy change regarding admission decisions that was implemented in October 2016. Before the policy change, consultations were mandatory for all patients requiring admission, as described in previous reports [13,16]. During and after the policy change, emergency physicians decided how to arrange for and treat medical patients by processing their admission and providing follow-up care without consultations. Consultations for specific procedures such as endoscopy or coronary procedures and consultations for expert opinions were continued before and after the policy change.

This study was approved by the Institutional Review Board (DAUHIRB-19-116). Written consent was waived by the committee because of the observational and noninvasive methodology of the study.

### 2.2. Study Setting and Population

The Dong-A University Hospital is located in Busan, a metropolitan city in the Republic of Korea. It is an academic tertiary-care hospital, and the annual ED census is approximately 32,000. The ED LOS and other indicators of patients admitted to the IM department were compared between the study period (January to June 2017) and the control period (January 2016 to June 2016). The study and control periods were selected to exclude the transition of practice that occurred at the end of 2016 and to avoid the effect of seasonal variation.

### 2.3. Measurements

The data that were extracted from the hospital information system database included the age, sex, ED LOS, admitted unit (either general ward or intensive care unit), admitted department and subspecialty, discharged department and division, hospital LOS, and the Korean Triage and Acuity Scale (KTAS) score from all the patients from the ED admitted to the IM department. KTAS is a modified assessment tool based on the Canadian Triage and Acuity Scale (CTAS) that was adapted for the Korean medical environment [21–23]. When the admitted department/division was different from the discharged department/division, the patient was considered an interdepartmental transfer. The total number of ED visits and ED LOS was also acquired to evaluate the impact of the policy change regarding IM-admitted patients on the overall ED LOS.

### 2.4. Data Analysis

The continuous data were presented as medians and interquartile ranges (IQRs) because the variables analyzed in the study, including age, appeared to have skewed distributions. The KTAS score was treated as an ordinal variable. The categorical data were summarized as frequencies (%). The continuous and ordinal data were compared using Wilcoxon's rank sum tests, and the categorical data were compared with chi-squared tests. *P* values below 0.05 were considered statistically significant. All analyses were conducted using R, version 3.6.1 (R Foundation for Statistical Computing, Vienna, Austria, 2019, available at http://www.R-project.org).

## 3. Results

**Figure 1:**
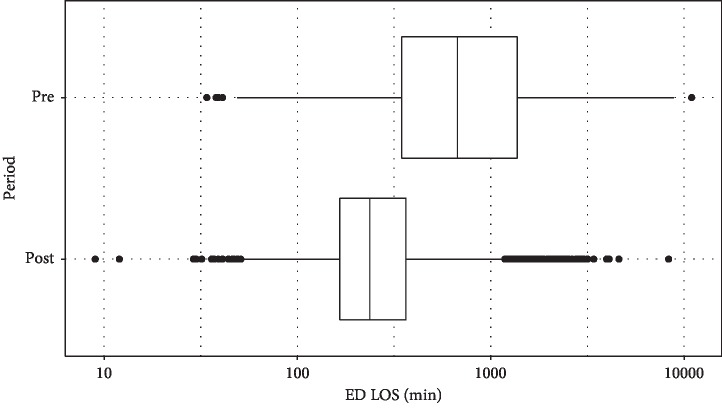
Emergency department length of stay of the patients admitted to the internal medicine department. The preperiod corresponds to January to June 2016, and the postperiod corresponds to January to June 2017. The central line indicates the median, the box represents the IQR, the whiskers extend to 1.5 times the IQR, and the point characters represent the outliers located beyond the whiskers. ED LOS: emergency department length of stay; IQR: interquartile range.

**Figure 2:**
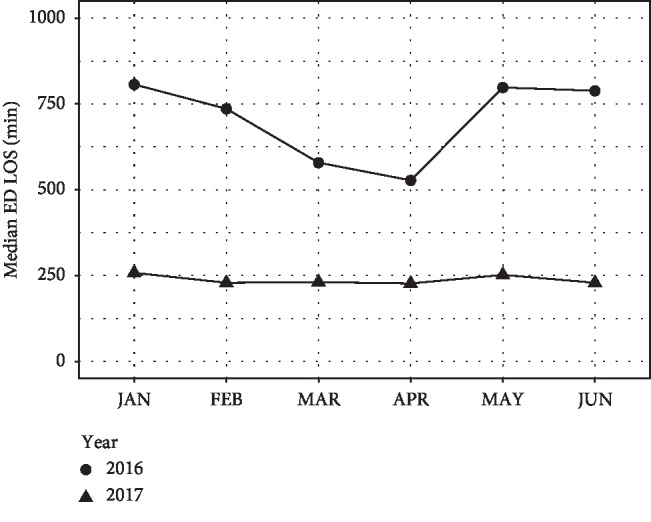
Trend in the emergency department length of stay of the patients admitted to the internal medicine department during the study period. ED LOS: emergency department length of stay.

A total of 15,326 patients visited the ED in the first half of 2016, and 15,031 visits were recorded during the same period in 2017. The characteristics of the general ED patients and ED workforce are summarized in Table 1. The total numbers of patients admitted to the IM department were 2,865 and 3,405 before and after policy change, respectively. The patient characteristics are summarized in Table 2. The acuity and severity represented by the KTAS score and ICU admission rate were not significantly different before and after the policy change. The median ED LOS of the patients admitted to the IM department decreased from 673 (IQR: 347–1,369) minutes in the control period to 237 (IQR: 166–364) minutes in the study period (*P* < 0.001, Figure 1).  Figure 2 shows the trend in the ED LOS of these patients that occurred during the study period. Although there was a statistically significant difference in the hospital LOS, the clinical significance of the difference was minimal, as the medians and IQRs were identical. There were no significant differences in the interdepartmental transfer rate or in-hospital mortality between the two periods. The overall median ED LOS decreased from 250 (IQR: 130–014) minutes before the policy change to 201 (IQR: 126–322) minutes after the policy change (*P* < 0.001).

## 4. Discussion

This study revealed that terminating the mandatory consultation policy reduced the ED LOS of admitted patients and thereby the overall ED LOS.

Howell et al. reported that admission decisions based on telephone consultations between ED physicians and in-house hospitalists rather than internal medicine residents reduced the ED LOS from 2.5 hours to 18 minutes [24]. Our results also suggest that admission decisions regarding medical patients made by EM physicians without mandatory consultations reduced the ED LOS of these patients and significantly decreased the overall ED LOS. Another study from Korea reported that the median ED LOS of patients admitted to some of the divisions in the IM decreased by 219 minutes after the mandatory consultation policy was abandoned [25]. The results are in agreement with those in the present study; however, the policy change in the previous study was limited to six divisions in the IM and the authors did not report the interdepartmental transfer rates [25].

There may be some concerns about the possible negative effects of eliminating specialty consultations. However, the in-hospital mortality and rate of interdepartmental transfers were not significantly different between the two periods in this study. Although the differences in age and hospital LOS were statistically significantly different between the study and control periods (*P* < 0.001), the medians and interquartile ranges were not considerably different between the periods. With a large sample size, statistical significance is likely to be present when there are no clinically significant differences; thus, in this study, the age and hospital LOS results should be interpreted accordingly [26]. Quick also found a similar result: after patient admission, the decision-making responsibility was shifted to the emergency physicians, the patients were stable when they arrived at the inpatient beds, and none of the patients were transferred to the ICU or to an operating room in the first 24 hours [27].

The present study found that the number of patients admitted to the IM department substantially increased from 2,865 to 3,405, while the total number of ED admissions slightly decreased. The trend was not apparent in the study by Choi et al. [25]. Although the acuity level of general ED patients measured by the KTAS appeared to be less severe in the postintervention period, the KTAS level of those admitted to the IM department was not significantly different between the two periods in this study. The rate of ICU admissions and APACHE II scores for those admitted to the ICU were also similar between periods, and these findings indicate that emergency physicians are not more likely to overtriage when issuing admission orders than their IM colleagues. The discrepancy between the periods in the numbers of IM admissions can be attributed to the longer preintervention ED LOS in our study. The prolonged observation period of patients in the ED seems to have been shifted to a stay in the inpatient admission department after the policy change. To decrease the ED LOS, observations should be performed by care units other than those in the ED. Researchers in New Zealand found that when the 6-hour mandatory ED LOS target was established by the government, the rate of use of short-stay units, acute assessment units, and observation wards within the ED or other parts of the hospital increased [26,28]. In our study, such units could not be used because of the shortage in the inpatient physicians, and it appears that the admission rate to regular wards increased.

There is concern that lack of consultations might have increased the burden of emergency physicians and negatively influenced the general ED performance. Although we did not measure the time to the first medical assessment, we compared the time to the first computerized physician order entry (CPOE) as a surrogate marker. The time to the first CPOE significantly decreased after the policy change (from 16.5 to 10.8 minutes), which can be explained by the decrease in ED overcrowding, positively influencing the timeliness of the ED. Moreover, while the burden of prolonged care for the patients increased, the burden of notifying and discussing issues with specialists in consultations decreased.

This study has several limitations. Because it was a retrospective observational study in a single center in Korea, the extent to which the results can be generalized to other centers with different situations and policies may be limited. Additionally, other changes in the way in which the hospital operates or the decrease in the overall number of ED visits that occurred during the one-year study period might have influenced the results. However, the reduction in the ED LOS was much more substantial in the population admitted to the IM department than the other ED patients. We could not control the effect of the severity of the patient conditions on the LOS and mortality. Although we found no difference in the KTAS score of the admitted patients between the two periods, the KTAS and the CTAS are used as initial triage tools rather than prognostic tools in the ED [21]. However, some studies have reported that the score has a strong association with prognoses, such as mortality and ICU admission rates [29,30].

## 5. Conclusions

Admission decisions regarding medical patients made by emergency physicians without specialty consultations reduced the ED LOS without a significant negative effect on mortality or hospital LOS.

## Figures and Tables

**Table 1:** Characteristics of the patients who visited the emergency department.

	January–June 2016	January–June 2017	*P* value
General demographics			
** **Total ED visit	15,326	15,031	
** **Males	8,325 (54.3)	8,213 (54.6)	0.575
** **Age (years)	53 (28–68)	55 (32–69)	<0.001
Time to the first CPOE (minutes)	16.5 (8.8–28.9)	10.8 (5.3–20.2)	<0.001
Acuity and severity			
** **Median KTAS score	3 (3–4)	4 (3–4)	<0.001
** **KTAS level 1 (immediate)	86 (0.6)	146 (1.0)	
** **KTAS level 2	1,345 (8.8)	1,283 (8.5)	
** **KTAS level 3	6,800 (44.4)	5,612 (37.3)	
** **KTAS level 4	6,303 (41.1)	6,697 (44.6)	
** **KTAS level 5 (may be delayed)	649 (4.2)	1,152 (7.7)	
** **Missing KTAS	143 (0.9)	141 (0.9)	
** **Total admission	5,957 (38.9)	6,432 (42.8)	<0.001
** **Admission to ICU	1,010 (16.9)	1,229 (19.0)	0.002
** **APACHE 2 scores of those admitted to the ICU	11.0 (7.0–18.0)	12.0 (7.0–18.0)	0.095
ED workforce and timeliness			
** **Faculty and attending physicians	5	6	
** **Residents	7	6	
** **Intern doctors	5	5	

ED: emergency department; CPOE: computerized physician order entry; KTAS: Korean Triage and Acuity Scale; APACHE: Acute Physiology and Chronic Health Evaluation Score; ICU: intensive care unit; LOS: length of stay. The data are represented by numbers (percentages) or medians (interquartile ranges), and the chi-squared test and the Wilcoxon's rank sum test were used to obtain the *P* values for the changes in the numbers and medians, respectively.

**Table 2:** Characteristics of the patients admitted to the medical department.

	January–June 2016	January–June 2017	*P* value
General demographics			
** **Admitted to internal medicine	2,865	3,405	
** **Males	1,598 (55.8)	1,883 (55.3)	0.706
** **Age (years)	66 (55–76)	68 (56–77)	<0.001
Acuity and severity			
** **Median KTAS score	3 (3–4)	3 (3–4)	0.694
** **KTAS level 1 (immediate)	8 (0.3)	26 (0.8)	
** **KTAS level 2	413 (14.4)	484 (14.2)	
** **KTAS level 3	1,670 (58.3)	1,991 (58.5)	
** **KTAS level 4	712 (24.9)	811 (23.8)	
** **KTAS level 5 (may be delayed)	62 (2.3)	91 (2.7)	
** **Missing KTAS	0 (0.0)	2 (0.1)	
** **Admitted to ICU	359 (12.5)	446 (13.1)	0.503
** **APACHE 2 scores of those admitted to ICU	15.0 (10.5–21.0)	15.0 (10.8–20.0)	0.755
Primary outcome			
** **ED LOS (minutes)	673 (347–1,369)	237 (166–364)	<0.001
Treatment quality variables			
** **Hospital LOS (days)	8 (5–14)	8 (5–14)	<0.001
** **Interdepartmental transfer after admission	185 (6.5)	254 (7.5)	0.121
** **In-hospital mortality	201 (7.0)	242 (7.1)	0.888

ED: emergency department; KTAS: Korean Triage and Acuity Scale; APACHE: Acute Physiology and Chronic Health Evaluation Score; ICU: intensive care unit; LOS: length of stay. The data are represented by numbers (percentages) or medians (interquartile ranges), and the chi-squared test and the Wilcoxon's rank sum test were used to obtain the *P* values for the changes in the numbers and medians, respectively.

## Data Availability

The data used to support the findings of this study have not been made available because of the relevant hospital policy.
